# Use of biologics in food allergy management 

**DOI:** 10.5414/ALX02141E

**Published:** 2021-02-19

**Authors:** Margitta Worm, Wojciech Francuzik, Sabine Dölle-Bierke, Aikaterina Alexiou

**Affiliations:** Division of Allergy and Immunology, Department of Dermatology, Venerology and Allergy, Charité – Universitätsmedizin Berlin, Germany

**Keywords:** food allergy, anti-IgE, oral tolerance induction, efficacy

## Abstract

Food allergies are a common medical problem, with children being the most affected patient group. The standard of care of food allergy consists of the acute treatment in case of a reaction and food avoidance in the long term, which influences the quality of life of patients. In this article, current developments for the causal treatment of food allergy including specific immunotherapy and biologics will be discussed. Epicutaneous and oral immunotherapy are currently in clinical development for the treatment of food allergy, and the results demonstrate good tolerability and efficacy with an increase in the oral threshold level. Biologics and, in particular, anti-IgE are currently investigated for their therapeutic use in food allergies. The results are promising, suggesting efficacy and tolerability.

## Introduction 

Food allergy is a common disease. It affects ~ 5 – 6% of children [1] and 2 – 3% of adults [2]. Symptoms of food allergy can be mild in the sense of an oral allergy syndrome, which is more common in adults in the context of pollen-associated food allergy, or can manifest as a systemic reaction in the sense of anaphylaxis. Data from the Anaphylaxis Registry show that in food-related anaphylaxis, skin symptoms are most frequent, followed by respiratory symptoms, while gastrointestinal symptoms are found in ~ 50% of affected individuals and cardiovascular symptoms in 40% [3]. An IgE-mediated mechanism underlies food allergy; mast cell-bound IgE is cross-linked upon ingestion of the allergen, leading to a release of the mast cell mediators, such as histamine, leukotrienes, and others, which in turn cause the clinical reactions. The treatment of food allergy includes acute therapeutic measures in the event of a reaction upon ingestion of the food, as well as allergen avoidance [4]. This leads to a burden on the quality of life of those affected. Due to accidental ingestion of food allergens, repeated reactions occur in up to 30% of food allergy patients. Although a fatal outcome of food allergy is a rare event, deaths, especially in childhood, have been reported [5]. 

These observations highlight the need for the development of therapeutic interventions that allow affected individuals to be protected in their daily lives while not compromising their quality of life. In contrast to inhalation allergies, specific immunotherapy has not been fully established for the treatment of food allergy to date. Reasons were the frequent onset of systemic reactions during the course of therapy in the first studies using subcutaneous administration [6, 7]. After many decades of little progress in the field of specific immunotherapy with food allergens, two treatment forms are currently in clinical development. One is the epicutaneous immunotherapy. Clinical trial results have demonstrated efficacy in terms of an increase of the oral threshold dose after a treatment period of several months, with very good tolerability [8]. Another way is the oral immunotherapy. This has been successfully performed in individual cases in allergy centers in selected patients for years [9]. The debate as to whether long-term oral tolerance or rather desensitization is achieved with this form of treatment has not yet been finally resolved. Not all patients experience long-term stable tolerance to the food in question after discontinuation of oral tolerance induction [10]. Recently, the efficacy and safety of peanut-specific oral immunotherapy has been evaluated in phase 2 and 3 clinical trials as well. Also these studies have shown clinical efficacy in terms of an increased tolerance to peanut protein amounts up to 1 g [11, 12]. However, systemic reactions occurred in up to 10% of patients, especially during the escalation phase of the treatment protocol. In this respect, this therapy should be exclusively performed in specialized centers under appropriate medical supervision. Moreover, the affected individuals and/or family members should be sufficiently educated regarding the emergency management in the event of a reaction. As of November 2020, approval procedures are currently underway for both forms of peanut immunotherapy in Europe to make this treatment available to patients. The results of the clinical trials were most promising for children and adolescents, therefore any approval of peanut immunotherapy will currently target the pediatric population. 

## Use of biologics for the treatment of food allergy 

**Figure 1:**
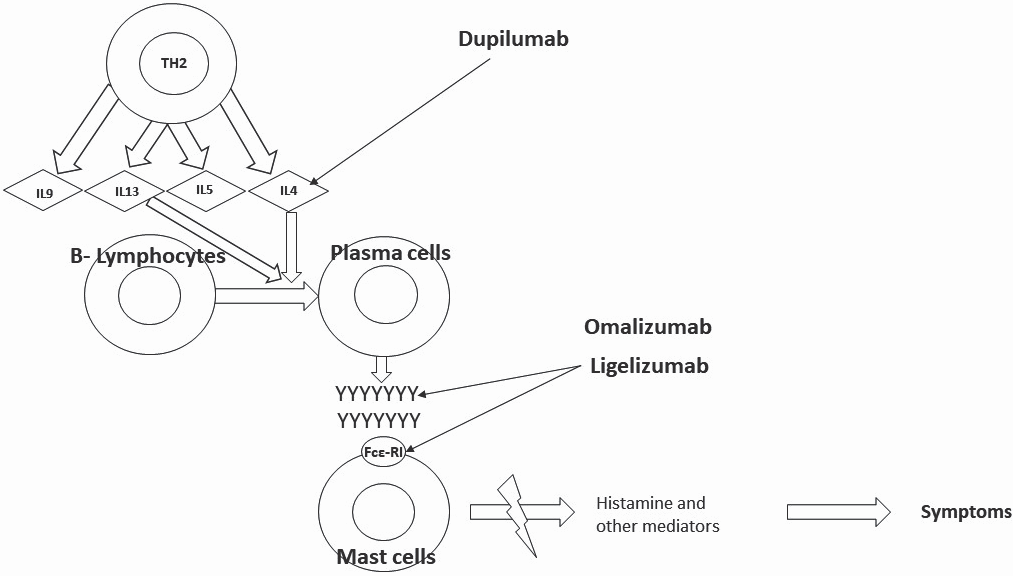
Use of biologics in food allergy. Y = IgEs, FcεRI = Fc epsilon receptor, MC = mast cells. Modified according to [24].

The longest-used biologic in allergy is the anti-IgE monoclonal antibody omalizumab, which has been approved for the treatment of steroid-resistant allergic asthma in Germany since 2005. Omalizumab (Figure 1) is a recombinant DNA-derived human IgG1 antibody that selectively binds IgE and prevents IgE binding to the high-affinity IgE receptor (FCεRI) on the surface of mast cells and basophils [13, 14]. The first study on the use of omalizumab in the context of specific immunotherapy was with a grass pollen extract [15]. Although the data did not show improved efficacy, it indicated better tolerability. The first study on the use of omalizumab in food allergy was published many years ago [16], but the development program at that time was not pursued for different reasons including the risk of severe reactions during therapy. 

Studies on the use of omalizumab for the treatment of food allergy were initiated again ~ 10 years ago. Here, the concept was to combine the potentially effective oral immunotherapy with omalizumab treatment to reduce the adverse reaction rate. Indeed, study results in peanut-allergic children show that it is possible to perform oral desensitization with peanut successfully and with a significantly reduced side effect profile. For example, it was shown in a study of 37 children treated with omalizumab for 12 weeks that a 1-day desensitization with up to 250 mg of peanut protein followed by weekly increases in peanut protein up to 2,000 mg was successful. 23 of the 29 patients treated with omalizumab (79%) tolerated 200 mg of peanut protein 6 weeks after omalizumab treatment ended, whereas only 1 of 8 patients (12%) in the placebo group achieved this. The onset of side effects rate was also significantly lower in the omalizumab-treated group. 

Omalizumab can also be used successfully in food allergy without combination with oral tolerance induction, as reported in numerous studies and individual case reports [16, 17, 18]. These studies reported an enhanced tolerance to peanut of 500 – 6,500 mg. Ultimately, the concept of monotherapy with omalizumab would require long-term treatment, whereas the combined use with oral immunotherapy would result in a transient biologic treatment. The latter could reduce long-term costs, and there would also be less injections with the risk of intolerance reactions. 

Another study investigated the efficacy of anti-IgE treatment in children allergic to multiple foods [19]. This study also demonstrated that at week 36, the omalizumab-treated group (30/36, 83%) was significantly more likely to tolerate 2 g protein for more than 2 of the food allergies compared with placebo (4/12, 33%) [19]. These data demonstrate that a treatment with omalizumab can also improve the efficacy of oral immunotherapy in patients with multiple food allergies (Table 1). A recent meta-analysis on the clinical efficacy of omalizumab in food allergy confirmed the great therapeutic potential of omalizumab [20]. Of 868 studies screened, 30 were included in this analysis. Of these, 8 were RCTs, 18 CCTs, and 4 were observational studies. In these studies, omalizumab as a monotherapy or as an adjunct to oral immunotherapy significantly increased the threshold levels for eliciting a clinical reaction to milk, egg, and in multifood-allergic patients compared to allergen-specific treatments alone. Moreover a significant impact on the quality of life in multifood allergic subjects was shown. Altogether, these data underline the importance of an anti-IgE based approach in food allergy. As the patent for omalizumab has expired 2018, the development of an anti-IgE biosimiliar is ongoing and will make this treatment easier accessible for more patients in the near future. 

Whether the second-generation anti-IgE ligelizumab will be even more effective for the treatment of food allergy in the future has to be evaluated in future clinical studies. First data from clinical phase 2 trials in chronic spontaneous urticaria suggested promising results [2^1^]. Ligelizumab shows better results in form of complete control of symptoms of chronic spontaneous urticaria with an acceptable safety profile. Patients treated with 3 different doses of Ligelizumab (24 mg, 72 mg, 240 mg) showed better results in complete control of disease (30%, 51%, and 42% respectively) in comparison to Omalizumab and placebo. 

In principle, an anti-IgE-based approach has the advantage to target at the same time other coexisting atopic diseases in a given patient, like allergic asthma or nasal polyps, the coverage of several food allergies if present, and the age-independent clinical efficacy. 

Another antibody of great interest for the treatment of food allergy is dupilumab. Dupilumab targets the IL-4 receptor α-chain and interferes with the IL-4 but also IL-13 signal transduction pathway (Figure 1). The antibody has been approved for the treatment of atopic dermatitis in Germany since 2017, and, in addition to very good clinical efficacy and tolerability, a decrease in total but also specific IgE was observed in patients during treatment so that efficacy in food allergy can also be suspected [22]. Clinical studies are currently being conducted in this regard, so that this interesting approach could also enable new therapeutic options in the future. 

## Conclusion 

Food allergy is a common medical condition which can lead to severe allergic reactions and may result, very rarely, even in fatal reactions. To date, there is no causal therapy for this disease so that avoidance of the triggering allergens is still considered the standard of therapy [4]. However, this not infrequently leads to a considerable reduction in the quality of life of those affected so that new therapies are urgently needed. Specific immunotherapy with food allergens is effective but associated with the not infrequent onset of systemic side effects. Therefore, there is a great potential for biologics, such as anti-IgE but also the anti-IL-4/IL-13 receptor antibody dupilumab, as they interfere with the IgE-dependent reactions present in food allergy in the long term. Studies are currently underway worldwide and will hopefully lead to a sustainable and safe therapeutic concept for food-allergic patients in the future. 

## Funding 

M.W. and S.D.-B. are supported by the German Research Foundation (KFO339). 

## Conflict of interest 

M.W. received honorarium for advisory boards and lecture activities from Regeneron Pharmaceuticals, DBV Technologies S.A, Stallergenes GmbH, HAL Allergie GmbH, Bencard Allergie GmbH, Allergopharma GmbH & Co. KG, ALK-Abelló Arzneimittel GmbH, Mylan Germany GmbH, Leo Pharma GmbH, Sanofi-Aventis Deutschland GmbH, Aimmune Therapeutics UK Limited, Actelion Pharmaceuticals Deutschland GmbH, Novartis AG, Biotest AG, AbbVie Deutschland GmbH & Co. KG and Lilly Deutschland GmbH. 

A.A. and S.D.-B. declare that there is no confict of interest. 

**Table 1.** Mechanisms and therapeutical concepts of omalizumab in food allergy.

Effect
– Blocks free IgEs
– Reduces cell-bound IgEs
– Reduces FcεR receptors
– Reduces release of mediators
Use in the treatment of food allergy
– Omalizumab combined with oral immunotherapy
– Omalizumab combined with multiple oral immunotherapies
– Omalizumab as monotherapy
Potential advantages
– Reduction in duration of therapy
– Better efficacy
– Better tolerability at higher doses
– Increased tolerance
– Reduction of the rate of side effects

Modified according to [23], [24].
